# Spc24 is required for meiotic kinetochore-microtubule attachment and production of euploid eggs

**DOI:** 10.18632/oncotarget.12453

**Published:** 2016-10-04

**Authors:** Teng Zhang, Yang Zhou, Hong-Hui Wang, Tie-Gang Meng, Lei Guo, Xue-Shan Ma, Wei Shen, Heide Schatten, Qing-Yuan Sun

**Affiliations:** ^1^ Institute of Reproductive Sciences, College of Animal Science and Technology, Qingdao Agricultural University, Qingdao, China; ^2^ State Key Laboratory of Stem Cell and Reproductive Biology, Institute of Zoology, Chinese Academy of Sciences, Beijing, China; ^3^ Department of Veterinary Pathobiology, University of Missouri, Columbia, MO, USA; ^4^ University of the Chinese Academy of Sciences, Beijing, China

**Keywords:** Spc24, meiosis, oocyte, kinetochore-microtubule attachment, aneuploidy

## Abstract

Mammalian oocytes are particularly error prone in chromosome segregation during two successive meiotic divisions. The proper kinetochore-microtubule attachment is a prerequisite for faithful chromosome segregation during meiosis. Here, we report that Spc24 localizes at the kinetochores during mouse oocyte meiosis. Depletion of Spc24 using specific siRNA injection caused defective kinetochore-microtubule attachments and chromosome misalignment, and accelerated the first meiosis by abrogating the kinetochore recruitment of spindle assembly checkpoint protein Mad2, leading to a high incidence of aneuploidy. Thus, Spc24 plays an important role in genomic stability maintenance during oocyte meiotic maturation.

## INTRODUCTION

Maintenance of genomic stability is a key prerequisite for all multicellular organisms. Abnormalities in chromosome number have been associated with human diseases, and maternally-derived aneuploidies are particularly problematic [1, 2]. Mammalian oocytes are error prone in segregating chromosomes during two successive meiotic divisions. Furthermore, most aneuploidies appear to be caused by mis-segregation of a bivalent in the first meiotic division [3, 4]. One of the most common viable aneuploidies is trisomy 21 resulting from mis-segregation of chromosome 21 during the first meiosis [2]. Therefore, there has been much interest in understanding how chromosome segregation is controlled in meiosis I.

During the first meiosis in oocytes, kinetochore-microtubule (K-MT) attachment is considered the key step for faithful chromosome segregation. Furthermore, the highly conserved KMN network (Ndc80, Mis12 and Knl1 complex) is primarily responsible for stable K-MT attachment and recruitment of the SAC (spindle assembly checkpoint) protein [5–8]. The inner end of the Ndc80 complex is anchored by the Mis12 complex, whereas the outer end is the primary binding site for the plus ends of spindle microtubules [7, 9–13]. Knl1 complex also binds, at its outer end, to spindle microtubules and, at its inner end, to the Mis12 complex [13]. The Aurora B kinase has a central role in regulating the stability of K-MT attachments and error attachment correction in concert with the KMN network [10, 14, 15]. We already know that Knl1 is required for recruiting several mitotic checkpoint proteins such as Bub1 and BubR1, and that Ndc80 is involved in recruiting Mad1 [16]. Eukaryotic cells employ the SAC mechanism to ensure accurate K-MT attachment and chromosome segregation during meiosis and mitosis.

Spc24 is one of the member of Ndc80 complex, which is consisted of two dimers, C-terminus of the Ndc80-Nuf2 dimer and the N-terminus of the Spc24-Spc25 dimer [16–18]. In vertebrate cells, Spc24 is associated with kinetochores from pro-metaphase through anaphase [19]. Knockdown of Spc24 using both RNAi or antibody injection showed that Spc24 is required not only for establishing, but also for maintaining K-MT interactions in mitotic cells [19]. In addition, Spc24 is required for chromosomal metaphase alignment and movement to the spindle poles [20]. Recently, in mouse oocytes, the Ndc80 complex has been reported to be important for meiotic progression. Depletion of one of Ndc80 complex proteins (Ndc80, Nuf2 or Spc25) caused chromosome misalignment and aneuploidy in mouse oocytes [21–23]. Overexpressed Nuf2 or Spc25 in mouse oocytes caused MI arrest and the SAC activation [21, 22]. Therefore, we reasoned that Spc24 may play a significant role in mouse oocyte meiosis.

Here, we demonstrate that Spc24 is indispensable for the recruitment of SAC proteins and kinetochore-microtubule attachment as well as euploidy maintenance in mouse oocytes.

## RESULTS

### Subcellular localization of Spc24 during mouse oocyte meiotic maturation

**Figure 1:**
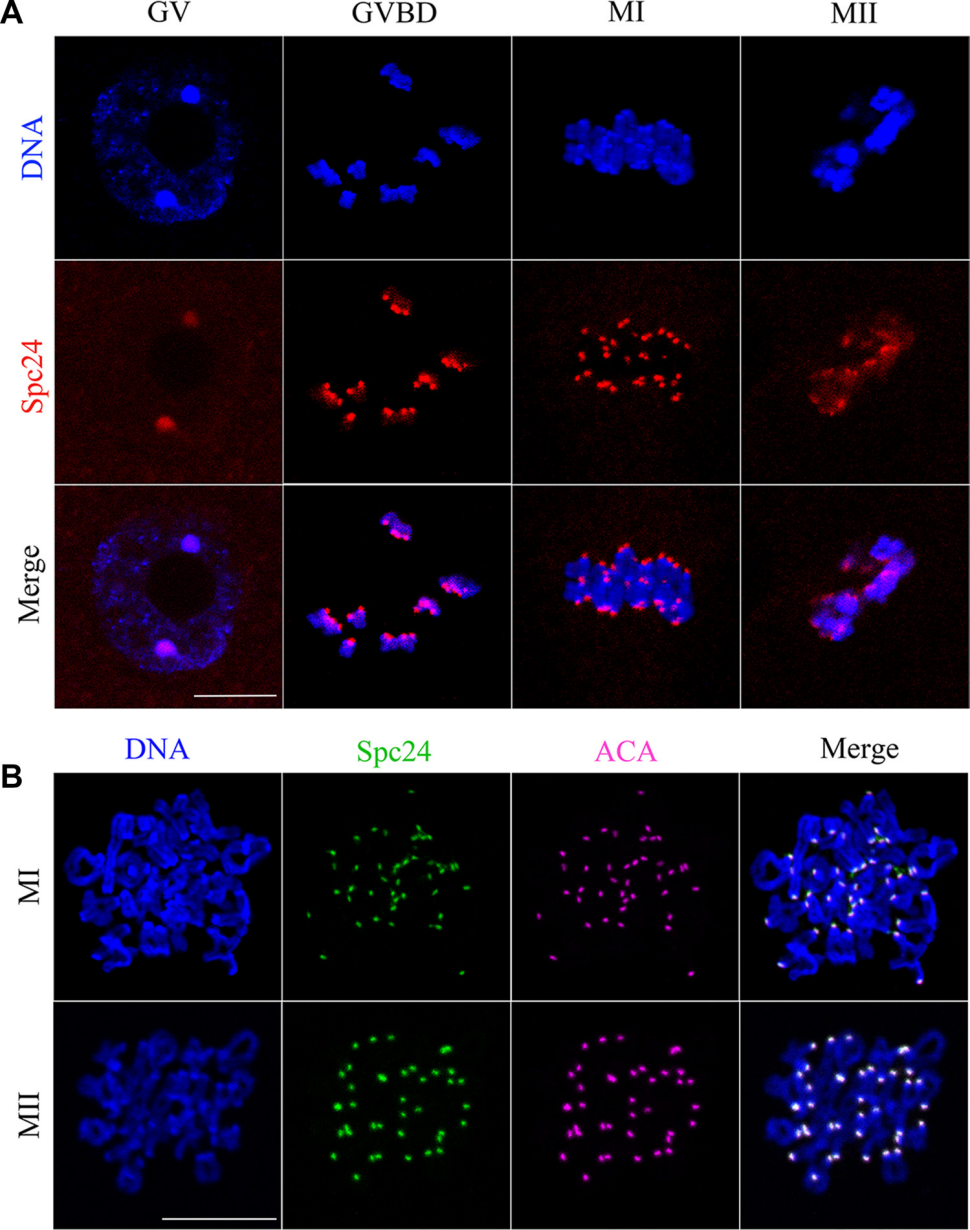
Subcellular localization of Spc24 during mouse oocyte meiotic maturation Oocytes were collected after culture for 0, 4, 8 and 12 h, the time points when most oocytes had reached the GV, GVBD, MI and MII stages, respectively. (**A**) Confocal microcopy showing the subcellular localization of Spc24 (red) in mouse oocytes at GV, GVBD, MI and MII stages. DNA (blue) was counterstained with Hoechst 33342. Scale bars: 20 μm. (**B**) The localization of Spc24 (green) and ACA (purple) on chromosome spreads at MI and MII stages. DNA (blue) was counterstained with Hoechst 33342. Scale bars: 20 μm.

To investigate the function of Spc24 during oocyte meiosis, we first examined localization of this protein using anti-Spc24 immunofluorescent staining. In the GV oocytes, Spc24 was primarily distributed in the germinal vesicles. Shortly after GVBD, the signal of Spc24 was observed at the kinetochores. When oocytes reached the MI and MII stages, clear staining was obvious at the kinetochores of chromosomes (Figure 1A).

To further confirm the localization of Spc24, ACA and Spc24 were double stained in chromosome spreads. As shown in Figure 1B, the signals of Spc24 completely overlapped with those of ACA at the MI and MII stages. It is revealed that Spc24 may involve in the K-MT attachment as a component of the kinetochore in mouse oocyte meiosis.

### Depletion of Spc24 accelerates passage through meiosis I

**Figure 2:**
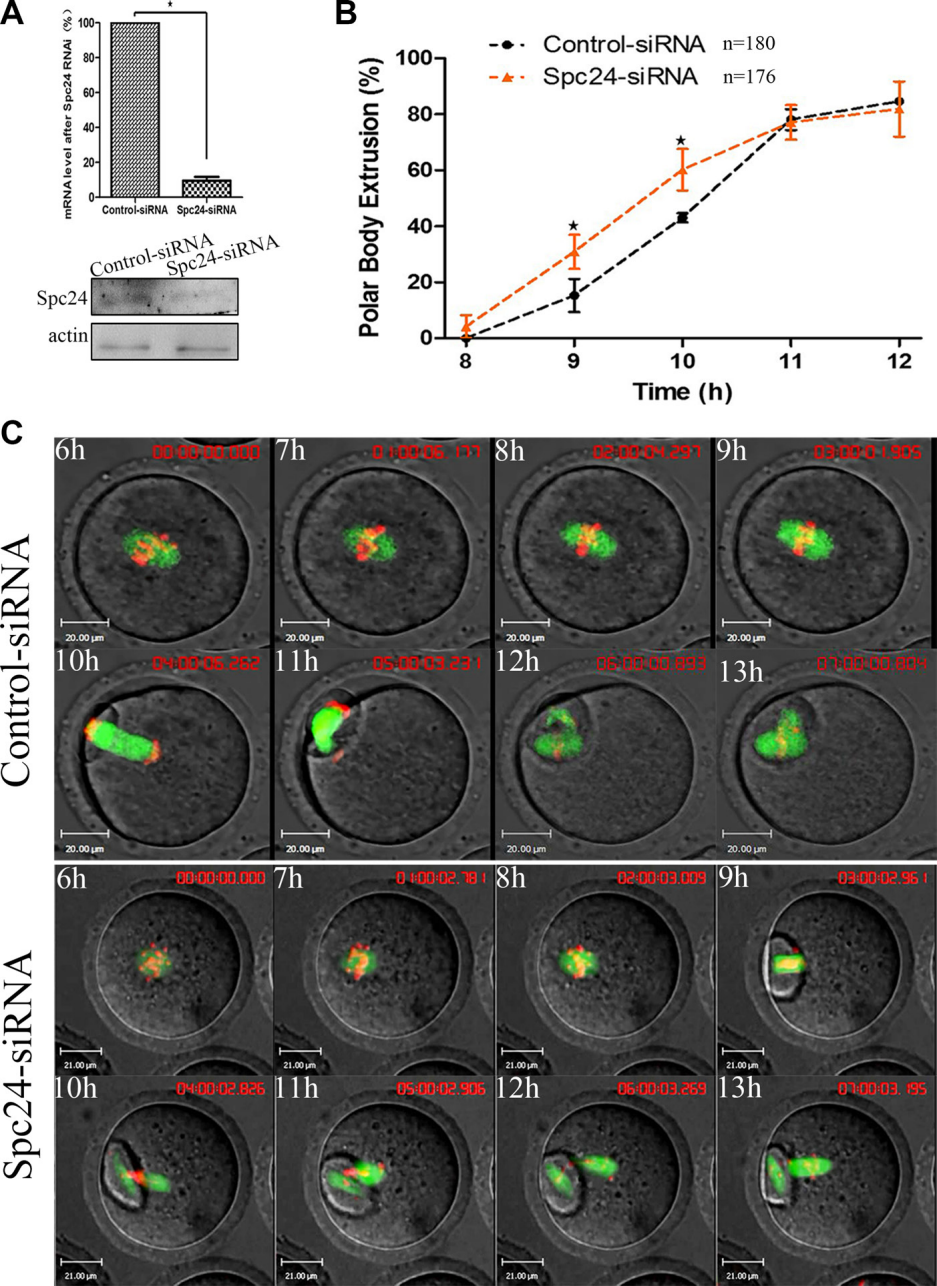
Knockdown of Spc24 accelerates polar body extrusion (**A**) Levels of Spc24 mRNA or protein in siRNA injected oocytes. The GV stage oocytes were microinjected with negative control siRNA or Spc24 siRNA and incubated for 24 h in M2 medium containing 200 μM IBMX before collecting the oocytes for real time quantitative PCR or western blot. (**B**) Timing of polar body extrusion was determined in Spc24-depleted oocytes and control oocytes. Data are expressed as mean ± SEM of at least 3 independent experiments. *Significantly different (*P* < 0.05). (**C**) Control or Spc24-depleted oocytes expressing α-tubulin-GFP and stained with Hoechst 33342 were visualized by time-lapse live-cell imaging. Time points indicate the time-lapse from about 3–4 h after GVBD. Note that the polar body extrusion was accelerated and chromosomes were misaligned in Spc24-depleted oocytes. α-tubulin (green); DNA (red). Scale bars: 20 μm. The total numbers of analyzed oocytes are indicated (n).

To determine the role of Spc24 in meiosis, we microinjected small interfering RNAs (siRNAs) targeting Spc24 into the GV stage oocytes and cultured them for 24 hours in the presence of IBMX to deplete this protein. Spc24 expression was significantly decreased after injection of Spc24 siRNA, implying successful Spc24 down-regulation by RNAi (Figure 2A). As shown in Figure 2B, Spc24-depleted oocytes underwent polar body extrusion, with no significant differences observed between control oocytes and siRNA injection oocytes. However, the timing of polar body extrusion was accelerated compared with the control oocytes at 9 h and 10 h (15.29 ± 8.37 vs. 30.86 ± 10.49%; 43.07 ± 2.79 vs. 60.23 ± 12.83%, *p* < 0.05), respectively, suggesting that depletion of Spc24 causes precocious polar body extrusion.

Next, live cell imaging was performed to detect the dynamic changes of chromosomes after injection of Spc24 siRNA. In the control oocytes, chromosomes aligned on the metaphase plate and migrated toward the oocyte cortex, followed by first polar body extrusion at about 11 h following release from IBMX. In contrast, Spc24-depleted oocytes underwent first polar body extrusion at about 9h following release from IBMX. These results suggest that knockdown of Spc24 results in precocious anaphase onset, followed by premature PB1 extrusion (Figure 2C).

### Spc24 is indispensable for recruitment of the spindle assembly checkpoint protein Mad2 to kinetochores

**Figure 3:**
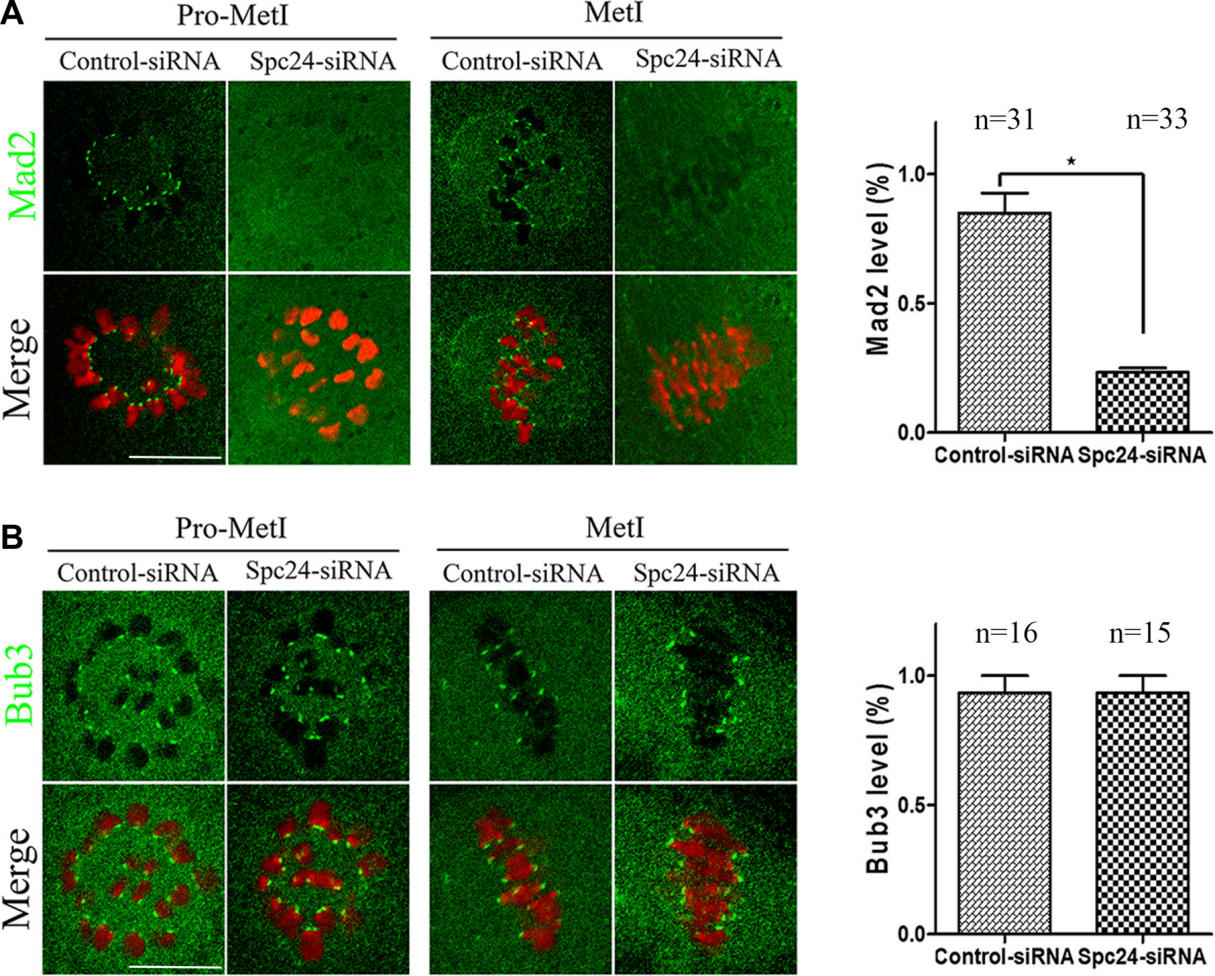
Mad2-mediated SAC inactivation in Spc24-depleted oocytes Control and Spc24-depleted oocytes were fixed at 5 h following release from IBMX. (**A**) Oocytes were immunostained with anti-Mad2 antibody (green) and Hoechst 33342 (red). Scale bars: 20 μm. (**B**) Oocytes were immunostained with anti-Bub3 antibody (green) and Hoechst 33342 (red). Scale bars: 20 μm. Quantification of fluorescent intensity of Mad2 or Bub3 is shown in the right panel of images, respectively. Data are expressed as mean ± SEM of at least 3 independent experiments. *Significantly different (*P* < 0.05). The total numbers of analyzed oocytes are indicated (n).

The precocious polar body extrusion implied that SAC activity was compromised in Spc24-depleted oocytes. To further confirm this possibility, the localizations of Mad2 and Bub3 were determined at 5 h following release from IBMX after Spc24 knockdown. Interestingly, Bub3 remained at kinetochores, while Mad2 no longer localized to kinetochores in Spc24-depleted oocytes (Figure 3A, 3B). Therefore, our results suggest that acceleration of meiosis I is due to a failure to recruit Mad2 at kinetochores in Spc24-depleted oocytes.

### Loss of Spc24 causes abnormal chromosome alignment and aneuploidy

**Figure 4:**
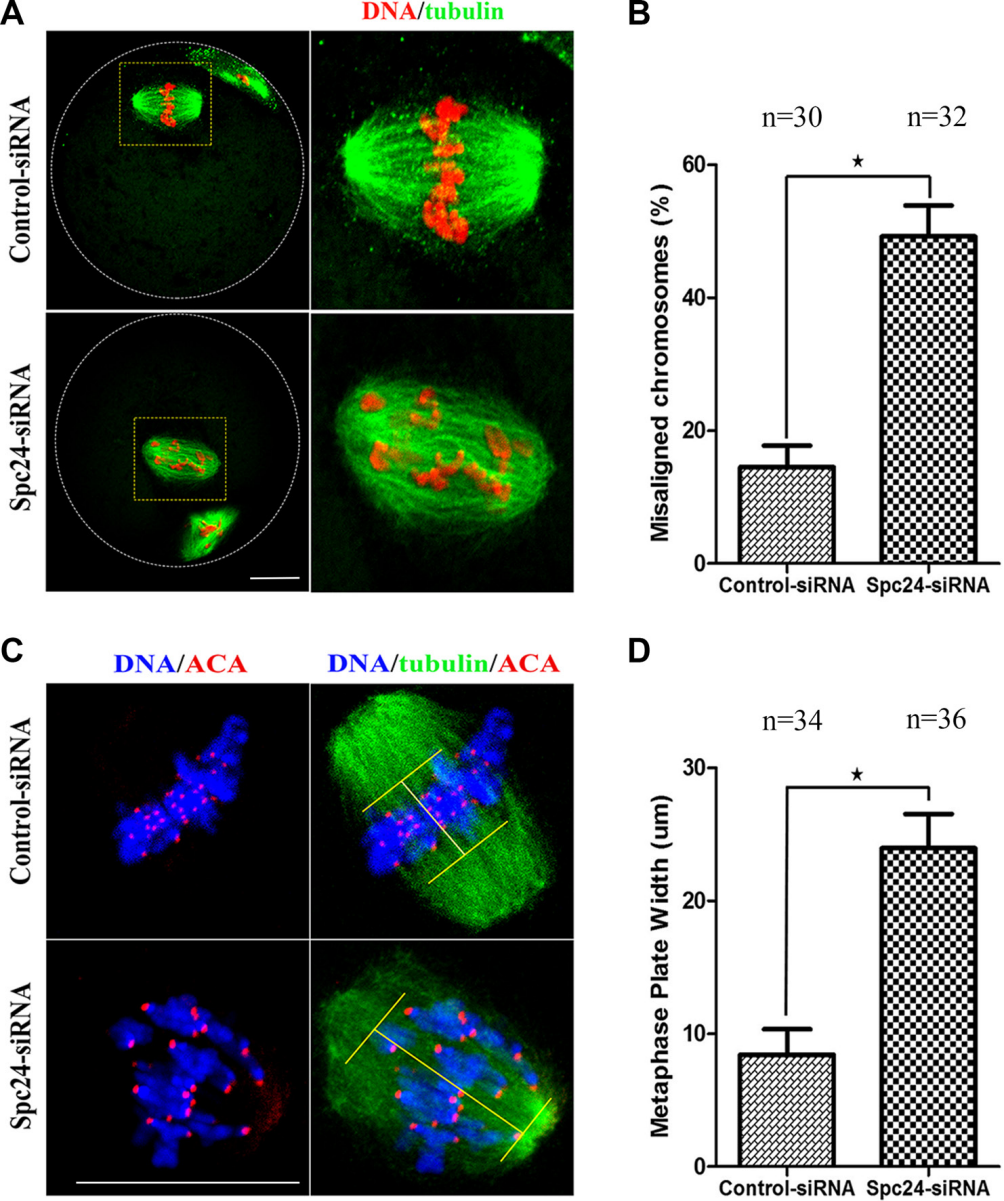
Loss of Spc24 causes misaligned chromosomes in meiotic oocytes (**A**) Abnormal chromosome alignment in MII oocytes after microinjection of Spc24 siRNA. In the control group, most oocytes showed normal chromosome alignment, while in the Spc24-depleted oocytes, most oocytes showed severely misaligned chromosomes. α-tubulin (green); DNA (red). Scale bars: 20 μm. (**B**) The rates of oocytes with misaligned chromosomes in the siRNA injection and control group. Data are expressed as mean ± SEM of at least 3 independent experiments. *Significantly different (*P* < 0.05). (**C**) Oocytes in MI were stained with anti-tubulin, ACA and Hoechst 33342. Scale bars: 20 μm. (**D**) Metaphase plate width was determined by measuring the axis distance between the two lines at the edges of the DNA. Data are expressed as mean ± SEM of at least 3 independent experiments. *Significantly different (*P* < 0.05). The total numbers of analyzed oocytes are indicated (n).

Considering the precocious anaphase onset, leading to an increase in the risk of aneuploidy [24], we hypothesized that depletion of Spc24 causes chromosome misalignment resulting in aneuploidy during oocyte meiosis. To test the hypothesis, the MII oocytes were cultured to investigate the chromosome alignment. The Spc24-depleted oocytes contained severely misaligned chromosomes compared with the control siRNA-injected oocytes (Figure 4A). As shown in Figure 4B, the rate of misaligned chromosomes in the Spc24 RNAi oocytes (49.23 ± 8.08%) and control oocytes (14.53 ± 5.54%) differed significantly (*P* < 0.05). After cultured of control oocytes for 8 h, chromosomes concentrated at the mid-plate (Figure 4C, 4D). However, Spc24-RNAi oocytes exhibited increased incidences of chromosome misalignments. Similarly, live-cell imaging showed that in Spc24-RNAi oocytes, various chromosomes were incapable of aligning at the middle plate at MI and MII stages (Figure 2C). Hence, it is suggested that loss of Spc24 leads to chromosome alignment disruption during the meiosis of mouse oocyte.

**Figure 5:**
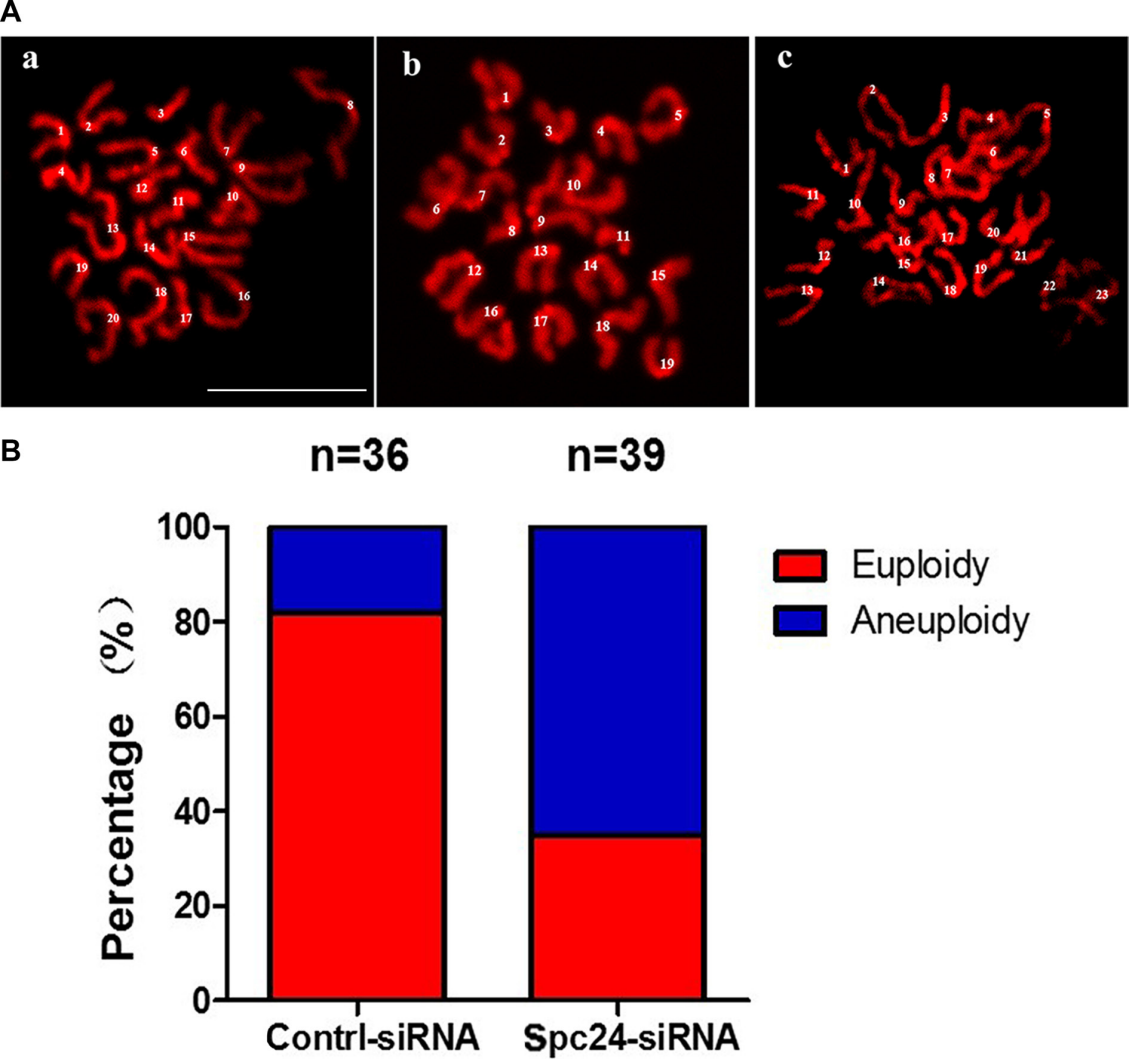
Knockdown of Spc24 causes aneuploidy (**A**) Oocytes in MII stage were collected and chromosome spreads were performed in control and Spc24 RNAi oocytes. Scale bars: 20 μm. (**B**) The numbers of univalents in the oocytes in A a; b; c are 20; 19; 23, (“a” is control oocyte, “b,c” are RNAi oocytes), respectively. The total numbers of analyzed oocytes are indicated (n).

To further confirm the effects of Spc24 siRNA injection, chromosome spreads were performed to show the pattern and number of chromosomes at the MII stage. As known for MII oocytes, the number of univalent chromosomes is 20. However, Spc24-RNAi oocytes typically displayed incorrect numbers of univalents. The numbers of chromosomes in the MII oocytes were 20, 19 and 23, respectively, confirming the Spc24-RNAi induced aneuploidy (Figure 5).

### Spc24 is required for accurate kinetochore-microtubule attachment

**Figure 6:**
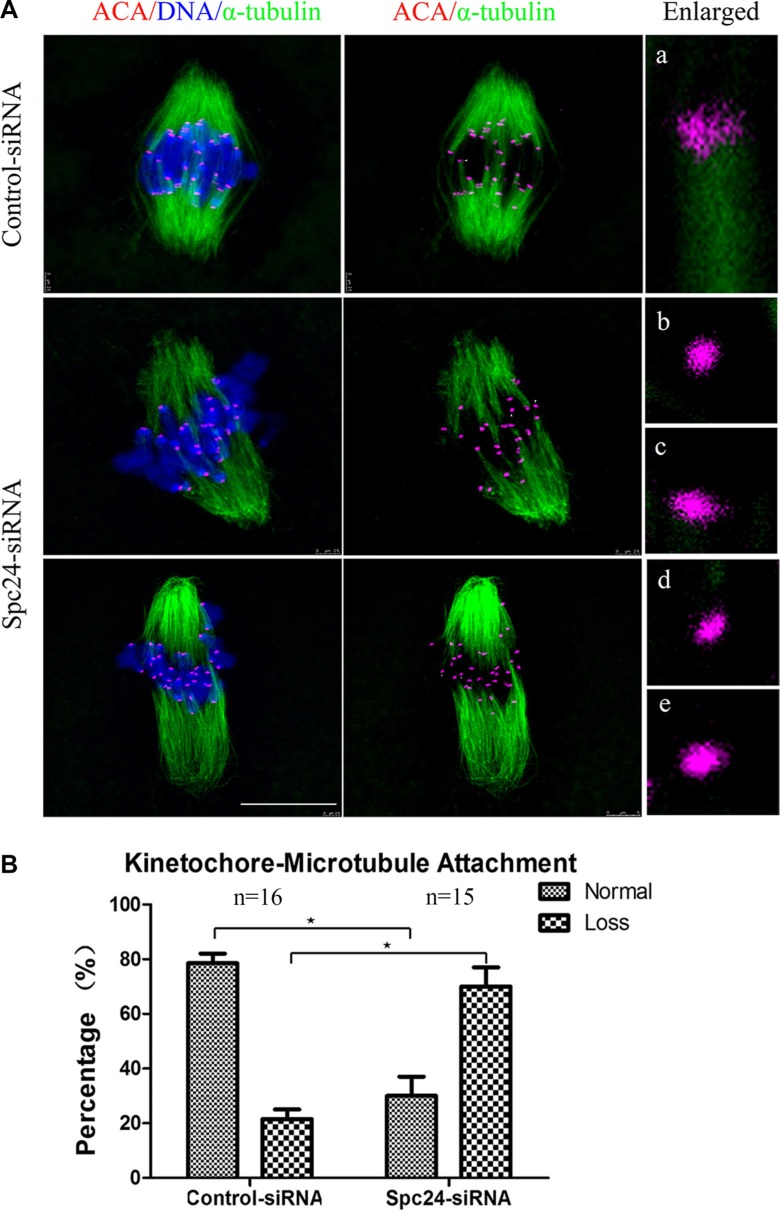
Correct kinetochore-microtubule attachment depends on Spc24 (**A**) Control and Spc24-depleted MI oocytes were cold-treated and stained with anti-tubulin (microtubules, green), ACA (kinetochores, purple), and Hoechst 33342 (chromosomes, blue). Enlarged views for the kinetochore-microtubule attachments in the right panel. Aa: normal attachment; Ab-e: loss of attachment. Scale bars: 10 μm. (**B**) Quantitative analysis of kinetochore-microtubule attachments in control and Spc24-depleted oocytes. Data are expressed as mean ± SEM of at least 3 independent experiments. *Significantly different (*P* < 0.05). The total numbers of analyzed oocytes are indicated (n).

Chromosome movement during oocyte meiosis involves a dynamic interaction between microtubules and kinetochores. Inappropriate K-MT attachment often results in misaligned chromosomes and aneuploidy in oocytes [25, 26]. To directly analyze the kinetochore-microtubule attachment status, we performed a brief cold treatment of oocytes at the MI stage. Different types of K-MT attachments were detected by confocal microscopy and categorized as normal attachment (each kinetochore attached to one of the poles; Figure 6Aa), and loss of attachment (kinetochores unattached to either pole; Figure 6Ab–e). These results demonstrated that the loss of attachment in Spc24-depleted oocytes was greatly increased compared to control oocytes, whereas the proportion of normal attachment was accordingly decreased (Figure 6B). Our results suggest that Spc24 is required for correct K-MT attachment, and disruption of Spc24 function resulting in mis-segregation of chromosomes and aneuploidy.

## DISCUSSION

Genomic stability of oocyte depends on accurate segregation of chromosomes, which requires dynamic association between kinetochores and microtubules. The correct K-MT attachment is a prerequisite for faithful chromosome segregation and for maintaining genomic integrity. Here, we demonstrated that Spc24 plays a pivotal role in faithful chromosome segregation by regulating K-MT attachment during mouse oocyte meiosis.

In mouse oocytes, the subcellular localization of Spc24 is not very different from that of mitosis [19]: the clear signal was obvious at the kinetochores at the meiotic metaphase (Figure 1A). The kinetochore localization of Spc24 completely overlapped with that of ACA at the meiotic metaphase stages (Figure 1B), suggesting that Spc24 may contribute to the surveillance of K-MT attachment during oocyte maturation. To characterize the functions of this protein, we knocked down the expression of Spc24 in GV stage oocytes by specific siRNA microinjection. We found that loss of Spc24 accelerated anaphase I onset, leading to precocious polar body extrusion (Figure 2B, 2C). Those oocytes may not undergo the prometaphase/metaphase arrest, but instead prematurely segregate chromosomes and enter anaphase. In oocyte meiosis, the SAC is thought to regulate the timing of polar body extrusion by regulating APC/C activity [27–29]. Previous studies also have shown that the Ndc80 complex regulates the SAC in mitosis and meiosis [30, 31]. Here, we showed that in the absence of Spc24, Mad2 failed to localize to kinetochores during meiosis, whereas the kinetochore localization of Bub3 was largely unaffected.

These results suggest that Spc24 selectively regulates the kinetochore recruitment of SAC proteins during meiosis, consistent with a previous report in somatic cells [30, 32, 33]. Indeed, oocytes with incorrect K-MT attachment can exist simultaneously with meiotic progression [34–36].

Precocious polar body extrusion may be caused by abnormal chromosome dynamics because oocytes may have undergone anaphase onset when chromosomes failed to align at the metaphase plate. In the present study, we showed that Spc24-depleted oocytes contained severely misaligned chromosomes at the meiotic metaphase (Figure 4). Similarly, live-cell imaging also showed that various chromosomes failed to align at the middle plate in Spc24-RNAi oocytes (Figure 2C). Escape of chromosomes from the equatorial plate at metaphase suggests that the interaction between kinetochores and the microtubules may have been disrupted, and microtubules failed to capture chromosomes. Attachment between microtubules and chromosomes depends on the kinetochore. The kinetochores provide the major microtubule attachment sites on chromosomes and thereby communicate forces generated by microtubule dynamics to power chromosome movement [37, 38]. Spc24, as a component of the kinetochore protein, plays a pivotal role in the correction of erroneous K-MT attachment in mitosis [19, 20]. In support of this notion, the K-MT attachment errors were readily observed in Spc24-depleted metaphase oocytes (Figure 6). These results collectively suggest that loss of Spc24 was able to disrupt the K-MT attachment, and thereby lead to misaligned chromosomes.

As it is known that chromosome segregation in female meiosis is notoriously error-prone, it is possible that oocytes enter anaphase without stable K-MT attachment. Most aneuploidies appear to be caused by the lack of faithful segregation of chromosomes during meiosis. Previous reports have indicated that erroneous K-MT attachment typically displayed incorrect numbers of univalents in oocyte meiosis [22, 25, 26]. In the present study, a high frequency of aneuploidy was detected in Spc24-depleted oocytes (Figure 5). These observations suggest that loss of Spc24 is one critical cause leading to aneuploidy by lacking of accurate K-MT attachment in oocytes. Furthermore, we provide evidence that such meiotic defects could dramatically increase aneuploidy.

In conclusion, our results reveal that Spc24 is indispensable for monitoring proper K-MT attachments and the recruitment of SAC proteins and thus chromosome fidelity in mouse oocyte maturation. Thus, Spc24 deficiency could have significant consequences for fertility by increasing aneuploidy in oocytes.

## MATERIALS AND METHODS

### Antibodies

Goat polyclonal anti-Spc24 antibody was purchased from Santa Cruz Biotechnology (Cat# sc-69241); Mouse monoclonal anti-α-tubulin-FITC antibody was obtained from Sigma-Aldrich Co (Cat# F2168,); Human polyclonal anti-ACA (anti-centromere antibody) antibody was purchased from Antibodies Incorporated (Cat# 15–234); Rabbit polyclonal anti-Mad2 antibody was purchased from Biolegend (Cat# 924601); Rabbit monoclonal anti-Bub3 antibody was purchased from Santa Cruz Biotechnology (Cat# sc-28258); FITC-conjugated goat anti-goat IgG and TRITC-conjugated goat anti-goat IgG were purchased from Zhongshan Golden Bridge Biotechnology Co, LTD.

### Oocyte collection and culture

Care and handing of 6–8 week-old ICR mice was conducted in accordance with policies promulgated by the Ethics Committee of the Institute of Zoology, Chinese Academy of Sciences. Oocytes were cultured in M2 medium supplemented with 200 μM 3-isobutyl-1-methylxanthine (IBMX) to maintain them at the germinal vesicle (GV) stage. After specific treatments, oocytes were washed thoroughly and cultured in M2 medium to specific stages.

### Microinjection of Spc24 siRNAs

Microinjection of siRNAs was performed as previously described using a Narishige microinjector and completed within 30 minutes. For knockdown experiments, small interfering RNAs (siRNAs) of Spc24 (GenePharma) were microinjected into the cytoplasm to deplete Spc24. The subsequent siRNAs were used at 20 μM, Spc24 siRNA-1: GGAGCUACGAGCAGAUGAUTT; AUCAUCUGCUCGUAGCUCCTT. Spc24 siRNA-2: GCUCAUCUCUAUCACCAAATT; UUUGGUGAUAG AGAUGAGCTT. The same amount of negative control siRNA was used as control. After microinjection, the GV oocytes were cultured for 24 hours in M2 medium supplemented with 200 μM IBMX for the depletion of Spc24.

### Real-time quantitative PCR analysis

Total RNA was extracted from 50 oocytes using RNeasy micro purification kit (Qiagen), the first strand cDNA was generated with M-MLV first strand cDNA synthesis kit (Invitrogen), using oligo (dT) primers. A cDNA fragment of Spc24 was amplified using the following primers: Forward: GACATGGTGGAGGTGAGCAA; Reverse: GGTGAC GGTGTTGTCCTCAT. GAPDH was selected as a reference gene using the following primers: Forward: TGGCAAAGTGGAGATTGTTGCC; Reverse: AAGATG GTGATGGGCTTCCCG.

### Immunofluorescence analysis

Immunofluorescent staining was performed as described previously [39, 40]. Oocytes were fixed in 4% paraformaldehyde in PBS buffer for 30 minutes at room temperature. After being permeabilized with 0.5% Triton X-100 for 20 minutes, they were then blocked in 1% BSA-supplemented PBS for 1 hour at room temperature. For staining of Spc24 or Mad2 or Bub3, oocytes were incubated overnight at 4°C with anti-Spc24 antibody (1:100); anti-Mad2 antibody (1:20); anti-Bub3 antibody (1:50), respectively. After three washes in washing buffer, oocytes were incubated with TRITC-conjugated goat anti-goat IgG (1:100) for 1 hours at room temperature. For α-tubulin staining, following incubation with anti-α-tubulin-FITC antibodies for 2 hours at room temperature, oocytes were washed 3 times in washing buffer, co-stained with Hoechst 33342 (10 mg/ml in PBS) for 15 min. These oocytes were mounted on glass slides and examined with a confocal laser-scanning microscope (Zeiss LSM 780 META, Germany).

### Chromosome spread

Chromosome spreads were performed as described previously [22]. Briefly, the oocytes were exposed to acid Tyrode's solution (Sigma) for 1–2 minutes at room temperature to remove the zona pellucida. After a brief recovery in M2 medium, the oocytes were transferred to glass slides and fixed in a solution of 1% paraformaldehyde in distilled H_2_O (pH 9.2) containing 0.15% Triton X-100 and 3 mM dithiothreitol. The slides were allowed to dry slowly in a humid chamber for several hours, and then blocked with 1% BSA in PBS for 1 hour at room temperature. The oocytes were then incubated with ACA (1:40), anti-Spc24 (1:100), overnight at 4°C. After brief washes with washing buffer, the slides were then incubated with the corresponding secondary antibodies for 2 hours at room temperature. The oocytes were finally stained with Hoechst 333342 after three washes in washing buffer and mounted on glass slides for immunofluorescence microscopy. The images were taken with a confocal laser-scanning microscope (Zeiss LSM 780, Germany).

### Cold treatment of oocytes

Spc24-depleted oocytes and control oocytes were first cultured for 8 h in M2 medium, and then transferred into M2 medium pre-cooled to 4°C and incubated in a refrigerator at 4°C for 15 min. Finally, these oocytes were collected and stained for immunofluorescent analysis with a-tubulin-FITC and ACA antibody [25]. The images were taken with a confocal laser-scanning microscope (Zeiss LSM 780, Germany).

### Time-lapse live-imaging experiments

After microinjecting Spc24 siRNA and α-tubulin-GFP mRNA, oocytes were incubated for 24 h in M2 medium supplemented with IBMX. Microtubule and chromosome dynamics were filmed on a Perkin Elmer precisely Ultra VIEW VOX Confocal Imaging System. A narrow band passed EGFP and BFP filter sets and a 30% cut neutral density filter from Chroma. Exposure time was set ranging between 300–800 ms depending on the α-tubulin-GFP and Hoechst 33342 fluorescence levels. The acquisition of digital time-lapse images was controlled by IP Lab (Scanalytics) or AQM6 (Andor/Kinetic-imaging) software packages. Confocal images of spindles and chromosomes in live oocytes were acquired with a 20× oil objective on a spinning disk confocal microscope (Perkin Elmer).

### Immunoblotting analysis

Immunoblotting was performed as described previously [40]. Briefly, the proteins were separated by SDS-PAGE and then transferred to PVDF membranes. Following transfer, the membranes were blocked in TBST containing 5% BSA for 2 hour at room temperature, followed by incubation overnight at 4°C with goat anti-Spc24 antibody (1:500) and mouse monoclonal anti-β-actin antibody (1:1,000). The membranes were incubated with 1:1000 HRP-conjugated goat anti-mouse or donkey anti-goat IgG, for 1 hour at 37°C. Finally, the membranes were processed using the enhanced chemiluminescence-detection system (Bio-Rad, CA).

### Image analysis

Images were acquired using a confocal laser-scanning microscope (LSM 780; Zeiss) equipped with a C-Apochromat 40× water immersion objective. Data analysis was performed using ZEN 2012 LSM software (Zeiss) and ImageJ software.

### Statistical analysis

Data (mean ± SEM) were generated from replicates that were repeated at least three times per experiment and analyzed by ANOVA using SPSS software (SPSS Inc., Chicago, IL) followed by student-Newman-Keuls test. Difference at *p* < 0.05 was considered to be statistically significant and different superscripts indicate the statistical difference.
